# Automated Quality Assessment of Structural Magnetic Resonance Brain Images Based on a Supervised Machine Learning Algorithm

**DOI:** 10.3389/fninf.2016.00052

**Published:** 2016-12-19

**Authors:** Ricardo A. Pizarro, Xi Cheng, Alan Barnett, Herve Lemaitre, Beth A. Verchinski, Aaron L. Goldman, Ena Xiao, Qian Luo, Karen F. Berman, Joseph H. Callicott, Daniel R. Weinberger, Venkata S. Mattay

**Affiliations:** ^1^Genes, Cognition, and Psychosis Program, National Institute of Mental Health, National Institutes of HealthBethesda, MD, USA; ^2^Department of Biomedical Engineering, UW-MadisonMadison, WI, USA; ^3^The Lieber Institute for Brain DevelopmentBaltimore, MD, USA; ^4^Bioinformatics and Computational Biosciences Branch, Office of Cyber Infrastructure and Computational Biology (OCICB), National Institute of Allergy and Infectious Diseases (NIAID), National Institutes of HealthRockville, MD, USA; ^5^NeuroImaging and Psychiatry, UMR 1000, Faculté de Médecine, Institut National de la Santé Et de la Recherche Médicale, Service Hospitalier Frédéric Joliot, Université Paris-SudOrsay, France; ^6^Behavioral Biology Branch, Walter Reed Army Research InstituteSilver Spring, MD, USA; ^7^Clinical and Translational Neuroscience Branch, National Institute of Mental Health, National Institutes of HealthBethesda, MD, USA; ^8^Departments of Psychiatry, Neurology and Neuroscience, Johns Hopkins University School of MedicineBaltimore, MD, USA; ^9^The Institute of Genetic Medicine, Johns Hopkins University School of MedicineBaltimore, MD, USA; ^10^Departments of Neurology and Radiology, Johns Hopkins University School of MedicineBaltimore, MD, USA

**Keywords:** structural magnetic resonance imaging, database management, automated quality assessment, machine learning, support vector machine, artifact detection, region of interest

## Abstract

High-resolution three-dimensional magnetic resonance imaging (3D-MRI) is being increasingly used to delineate morphological changes underlying neuropsychiatric disorders. Unfortunately, artifacts frequently compromise the utility of 3D-MRI yielding irreproducible results, from both type I and type II errors. It is therefore critical to screen 3D-MRIs for artifacts before use. Currently, quality assessment involves slice-wise visual inspection of 3D-MRI volumes, a procedure that is both subjective and time consuming. Automating the quality rating of 3D-MRI could improve the efficiency and reproducibility of the procedure. The present study is one of the first efforts to apply a support vector machine (SVM) algorithm in the quality assessment of structural brain images, using global and region of interest (ROI) automated image quality features developed in-house. SVM is a supervised machine-learning algorithm that can predict the category of test datasets based on the knowledge acquired from a learning dataset. The performance (accuracy) of the automated SVM approach was assessed, by comparing the SVM-predicted quality labels to investigator-determined quality labels. The accuracy for classifying 1457 3D-MRI volumes from our database using the SVM approach is around 80%. These results are promising and illustrate the possibility of using SVM as an automated quality assessment tool for 3D-MRI.

## 1. Introduction

High-resolution T1-weighted structural three-dimensional brain magnetic resonance imaging (3D-MRI^1^) is being increasingly used to assess brain morphological changes underlying neuropsychiatric disorders such as Parkinson's disease, Alzheimer's disease, and schizophrenia (Goldman et al., 2009; Jubault et al., 2011; Sabuncu et al., 2011). Unfortunately, image artifacts can compromise the utility of 3D-MRI volumes in brain morphometric studies. These artifacts include: (1) a rippling appearance in brain regions behind the orbits resulting from eye movement during acquisition, (2) a broad wave-like pattern near the top of the head resulting from motion induced ghosting of the bright fat in the skull, and (3) an aliasing artifact resulting from the field of view that is smaller than the object, whereby the nose and other facial structures appear overlaid on the posterior structures of the brain. Failure to exclude 3D-MRIs with such image artifacts frequently causes automated morphometric analysis routines to misclassify brain tissue type. This error can then be propagated into subsequent analyses involving gray matter intensity, shape, or surface, leading to spurious results. It is therefore important to screen 3D-MRIs for artifacts prior to using them in morphometric analyses. Thus far, there are very few studies in the literature that directly explored how image quality can affect identification of neuropathology from 3D-MRI (Magnotta et al., 2006; Woodard and Carley-Spencer, 2006).

Currently, the primary method to assess the quality of 3D-MRI is visual inspection, which can be subjective. Gardner et al. (1995) showed that human observers demonstrated poor sensitivity when evaluating intentionally degraded 3D-MRI volumes, as opposed to an automated approach, which detected even minimal noise in the images. One approach to reduce the workload of visually inspecting large number of 3D-MRIs is parallel processing by multiple investigators, such that each investigator inspects a subset of the data. However, such an approach can be unreliable, as each investigator uses a different threshold for accepting or excluding data. Additionally, this approach is time consuming, which makes the task of maintaining and updating the image quality information of large growing 3D-MRI datasets in a timely manner challenging. Therefore, automating the image quality rating procedure can improve the reproducibility, reliability, and efficiency of morphometric analysis, particularly when maintaining large neuroimaging databases (Cheng et al., 2009).

Mortamet et al. (2009) implemented a univariate and automated approach using two quality indices to assess the quality of 3D-MRIs. Three issues limit this univariate approach. First, a single metric cannot characterize artifacts coming from multiple sources. To accurately characterize artifacts from multiple sources, dedicated features need to be extracted, and mapped onto a multi-dimensional space. Univariate classification methods cannot take multiple features as inputs, nor can they form non-linear classification boundaries. These limitations can introduce classification errors near the boundary. Second, a single global volumetric metric can be limited in characterizing artifacts arising from small localized regions within a 3D-MRI volume, as global metrics are not sensitive to the location of the artifact. For instance, 3D-MRI volumes with similar artifact levels located in distinct regions of importance would result in similar ratings with a univariate approach. Third, when classified as “not-usable,” it would be helpful to have details on the type and location of artifacts present in the image, information that is attainable with a multivariate approach. It is possible that a 3D-MRI can be classified “not-usable,” based on global metrics but still can be “usable” for a region of interest (ROI) analysis. Therefore, a single global metric is often inadequate to optimally assess how a 3D-MRI volume is rendered “not-usable.”

We investigated the utility of a machine-learning algorithm on multi-dimensional, non-linear classification to overcome errors that can arise from a univariate approach. Specifically, the application of a supervised classification algorithm based on a support vector machine (SVM) (Burges, 1998; Vapnik, 2013) was explored to automatically rate the quality of 3D-MRI. The classification procedure was implemented in two stages. During the first stage, six different types of features were extracted from 1457 3D-MRI volumes. A 10-fold cross validation technique was used to separate these 3D-MRI volumes into a training and a testing subset. Different combinations of the extracted features of the training subset were used to train the SVM algorithm. In the second stage, the trained SVM algorithm was used to assess the quality of a testing subset, by using the features computed in the first stage. Classification accuracies were computed by comparing the classification results obtained using the SVM approach to that based on investigator-based visual inspection. The advantages, limitations, and possible future improvements of the current approach are discussed.

## 2. Materials and methods

**Figure 1:**
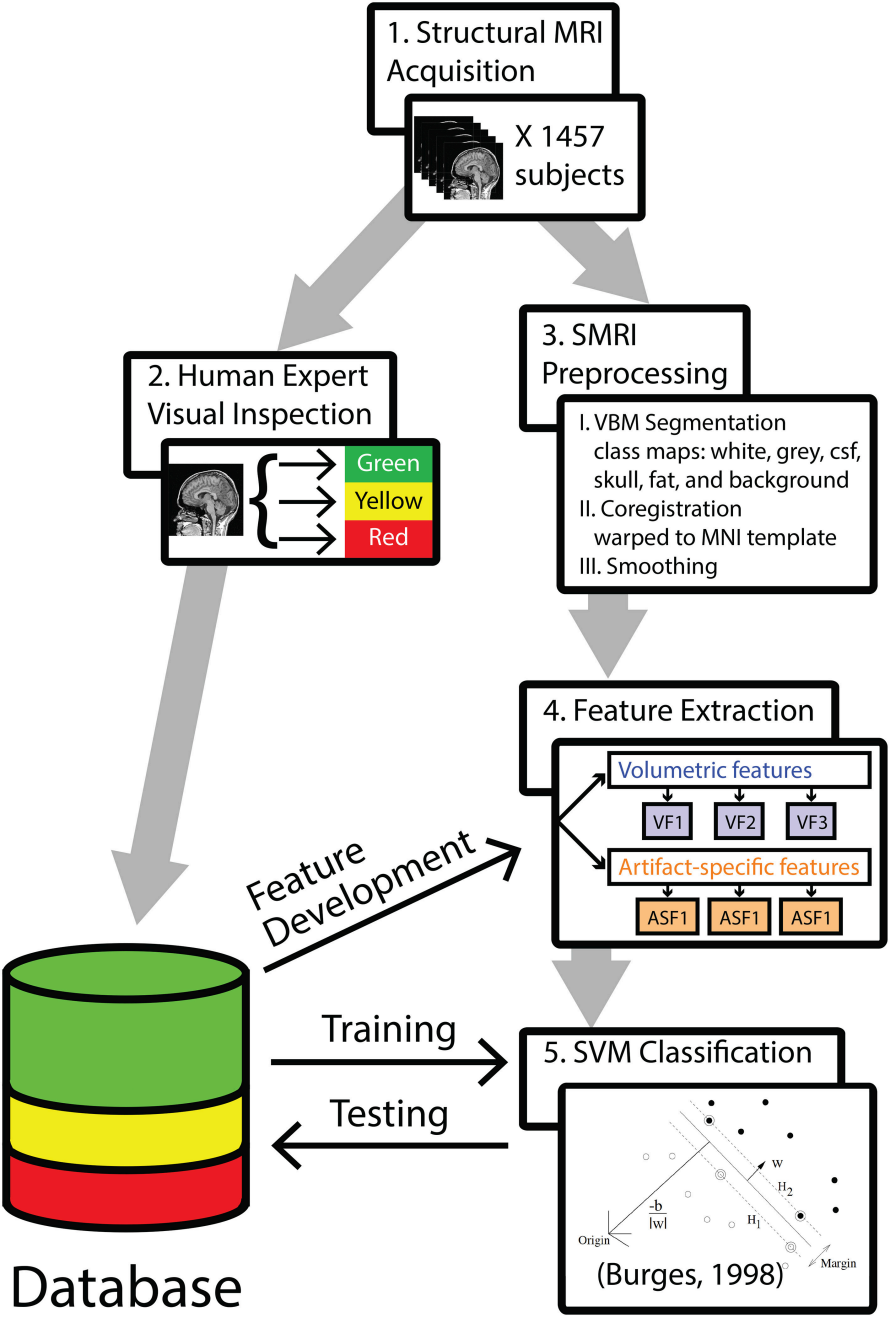
**A methods flowchart is presented to illustrate an overview of the steps involved in classifying structural 3D-MRIs in an automated fashion**.

The analysis pipeline is outlined in Figure 1. In step 1, 1457 T1-weighted 3D-MRIs were retrieved from our group's database (acquisition details are provided in Supplemental Material). In step 2, investigators visually inspected the 3D-MRI volumes. Each 3D-MRI was preprocessed in step 3, in order to extract features in step 4. In step 5, the SVM was used to classify the 3D-MRIs. The performance of SVM-based classification was evaluated by comparing to the visual inspection procedure.

### 2.1. Visual inspection, a reference point of data quality

**Figure 2:**
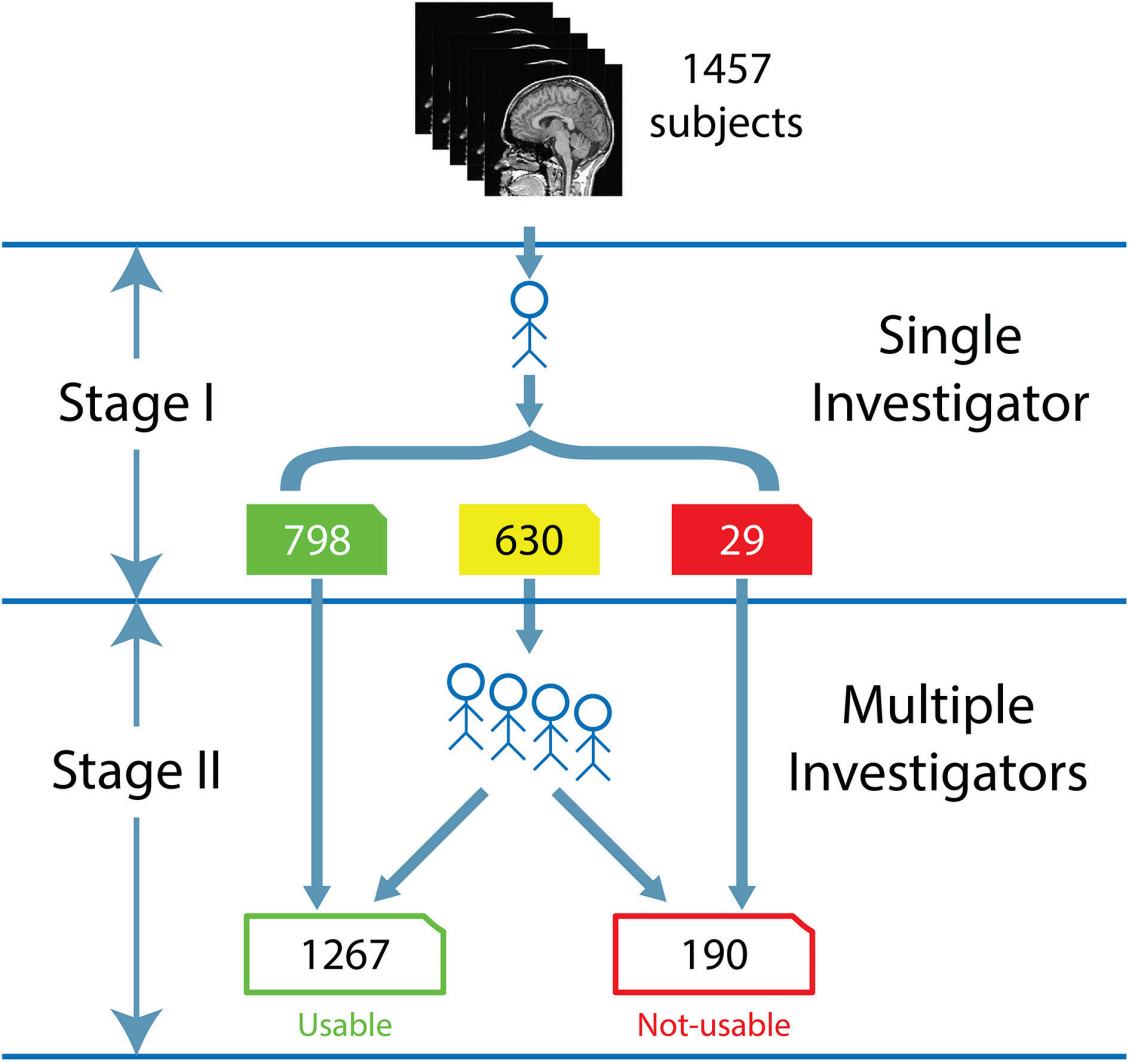
**The flow for the human visual inspection procedure was realized for all 1457 datasets, is illustrated above and comprises of 2 Stages:** (I) Visual inspection is performed by a single investigator to label the 3D-MRI volumes as green, yellow, or red. (II) Five to nine investigators then meet to further categorize the yellow 3D-MRI volumes as either usable or not-usable.

**Figure 3:**
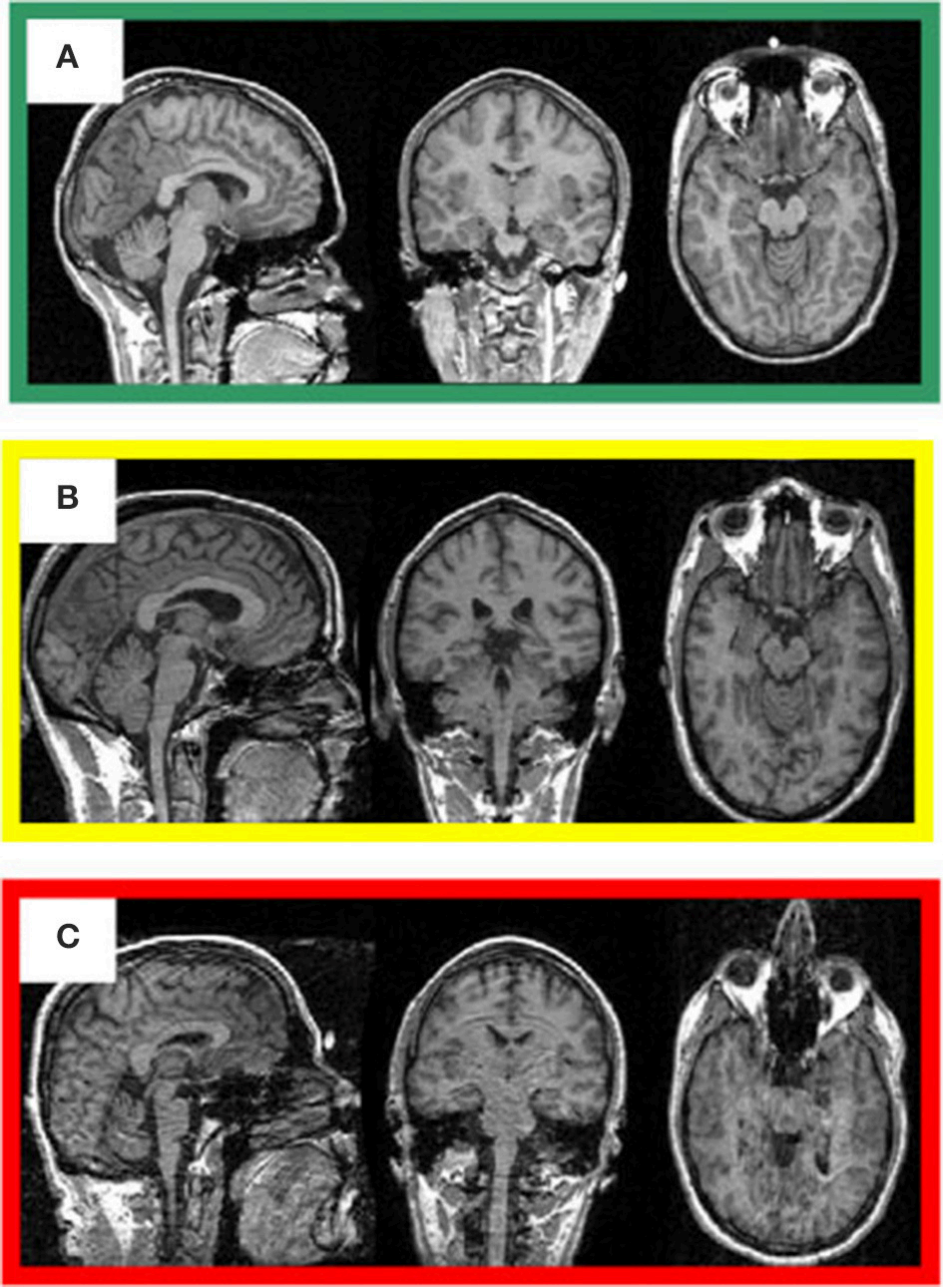
**Representative 3D-MRI volumes are presented with corresponding image quality**. **(A)** Green indicates usable and has excellent contrast between gray and white matter. **(B)** Volume contains slight ringing, was labeled yellow at stage I and usable at stage II. **(C)** Red indicates not-usable, has ringing, eye, and head movement.

The visual inspection procedure was performed in two stages as shown in Figure 2. In Stage I, the 3D-MRI visual inspection task was distributed among a group of investigators, such that each investigator was assigned a subset of 3D-MRIs for initial inspection. Investigators searched each slice for recurring artifacts, such as eye movement, ringing, aliasing, grainy images, head movement, and teeth filling. The severity of each artifact was evaluated based on a 4° rating scale: heavy, moderate, slight, or none. These descriptions provide details for the visual classification of 3D-MRIs. If the investigator deemed that all slices were of good quality with high contrast between gray and white matter, and contained no noticeable artifacts, then the 3D-MRI volume was labeled green. If the investigator deemed that artifacts compromised any brain region and the slice was clearly bad, the 3D-MRI volume was labeled red. Otherwise, the 3D-MRI volume was labeled yellow, indicating the 3D-MRI contained slight to moderate level artifacts. After Stage I, there were 798 green 3D-MRIs, 630 yellow 3D-MRIs, and 29 red 3D-MRIs. Typical examples are shown in Figure 3.

In Stage II, the green and red 3D-MRI volumes were labeled “usable” and “not-usable,” respectively. A group of 5–9 investigators further classified the yellow 3D-MRI volumes as “usable” or “not-usable” based on a majority vote. At the end of Stage II, the 1457 3D-MRI volumes of the dataset were classified by the visual inspection into 1267 “usable” 3D-MRIs and 190 “not-usable” 3D-MRIs. The final evaluation was used as the reference point in order to train and assess the performance of the SVM classifier.

### 2.2. 3D-MRI preprocessing

In the remainder of the Methods, *I*_*w,i*_(*x, y, z*) denotes the intensity at coordinates (*x, y, z*), in the acquired 3D-MRI *w*, taken from an individual subject *i*. All 1457 3D-MRIs **I**_*w,i*_, were preprocessed using the voxel-based morphometry (VBM) toolbox, as implemented in statistical parametric mapping (SPM8) software (Ashburner and Friston, 2000; Friston et al., 2003). Briefly, a unified segmentation (Ashburner and Friston, 2005) approach was used for tissue classification, image registration, and non-uniformity image intensity corrections. The tissue classification procedure generated six tissue probability maps from each **I**_*w,i*_, denoted as **p**_*c,i*_, where *c* = { gray matter, white matter, cerebral spinal fluid (CSF), skull, fat, and background }. The image registration procedure generated a transformation matrix *H*_*i*_ for each subject *i* to spatially normalize each 3D-MRI **I**_*w,i*_, from native (*x, y, z*) to normalized space (*x*′, *y*′, *z*′). In addition, an inverse transformation matrix $H_{i}^{-1}$ was generated, to map from the normalized space (*x*′, *y*′, *z*′) back to native space (*x, y, z*).**I**_*w,i*_, *H_i_*, $H_{i}^{-1}$, and **p**_*c,i*_, were used to extract the features used for the automated classification.

### 2.3. Feature extraction

The accuracy of the classification depends on the features used; hence, defining features is a crucial step in SVM classification. These features fall into two broad categories: volumetric features and artifact-specific features. After the 1457 3D-MRI volumes were preprocessed, these features were computed and used in the SVM classifier algorithm.

#### 2.3.1. Volumetric features (VF)

First, three different features were computed to quantify the artifacts in each 3D-MRI. The initial goal was to define a volumetric measure that quantifies artifacts throughout the 3D-MRI volume, similar to Mortamet et al. (2009). Three different features were developed to quantify the contrast between gray and white matter, motivated by the fact that good “usable” 3D-MRI should have high contrast between these two tissue types when compared to noisy 3D-MRIs, where contrast is lower.

##### 2.3.1.1. VF1—3D-MRI histograms

A histogram of the intensity values over each 3D-MRI was computed. The histogram of a 3D-MRI has profiles with peaks representing the gray matter, white matter, cerebral spinal fluid (CSF), skull, fat, and background. We expected high contrast 3D-MRIs to have two well-defined and distinct gray matter and white matter profiles over the histogram, and a low contrast 3D-MRIs to have indistinguishable gray matter and white matter voxels resulting in overlapping profiles in the histogram.

The 3D-MRI histogram, **VF1**, was computed as follows. Given the intensity value of the native space 3D-MRI **I**_*w,i*_, the range of the intensity [0, *I*_*max*_] was divided into 100 bins of width Δ_1_ = *I*_*max*_/100, where $I_{max}=\underset{x,y,z}{\max}I_{w,i}\left(x,y,z\right)$. This resulted in the bins of the histogram being centered on intensity steps $I_{n}=\left\{\frac{\Delta_{1}}{2},\frac{3\Delta_{1}}{2},\frac{5\Delta_{1}}{2},\ldots ,I_{max}-\frac{3\Delta_{1}}{2},I_{max}-\frac{\Delta_{1}}{2}\right\}$. The number of voxels for each bin *I*_*n*_ was then computed as:

(1) $$\begin{array}{rc} VF1\left(I_{n}\right) & =\underset{x,y,z}{\sum }\left[I_{n}-\frac{\Delta_{1}}{2}<I_{w,i}\left(x,y,z\right)\leq I_{n}+\frac{\Delta_{1}}{2}\right] \end{array}$$

Each value *VF*1(*I_n_*) represents the number of voxels that have an intensity value in the range defined in Equation (1), i.e., a histogram. For each subject, the vector **VF1**, of length 100 and equivalent to the entire histogram was used as features into SVM.

##### 2.3.1.2. VF2—class map histograms

Three class probability maps, **p**_*c,i*_, where *c* = {white matter, gray matter, CSF}, were used to create class map histograms as features. We expected high contrast 3D-MRIs to have more well-defined voxels with higher probabilities of belonging to a particular class, and low contrast 3D-MRIs to have more indistinguishable voxels with similar probabilities of belonging to a particular tissue type.

The class map histogram, **VF2_c_**, was computed for all 1457 3D-MRIs as follows. Given **p**_*c,i*_, the probability range [0, *p*_*max*_] was divided into 100 bins of size Δ_2_ = *p*_*max*_/100, where *p*_*max*_ = 1. This resulted in the bins of the histogram being centered on a wide range of probabilities $p_{n}=\;\left\{\frac{\Delta_{2}}{2},\frac{3\Delta_{2}}{2},\frac{5\Delta_{2}}{2},\ldots ,p_{max}-\frac{3\Delta_{2}}{2},p_{max}-\frac{\Delta_{2}}{2}\right\}$. The number of voxels for each bin *p*_*n*_ was then computed as:

(2) $$\begin{array}{rc} VF2_{c}\left(p_{n}\right) & =\underset{x,y,z}{\sum }\left[p_{n}-\frac{\Delta_{2}}{2}<p_{c,i}\left(x,y,z\right)\leq p_{n}+\frac{\Delta_{2}}{2}\right] \end{array}$$

Each value *VF*2_*c*_(*p_n_*) represents the number of voxels that have a probability value in the range defined in Equation (2), i.e., a histogram. For each subject, the vector **VF2_c_**, each of length 100 and equivalent to the entire class histogram was used as features into SVM.

##### 2.3.1.3. VF3—gw_t_score

**Figure 4:**
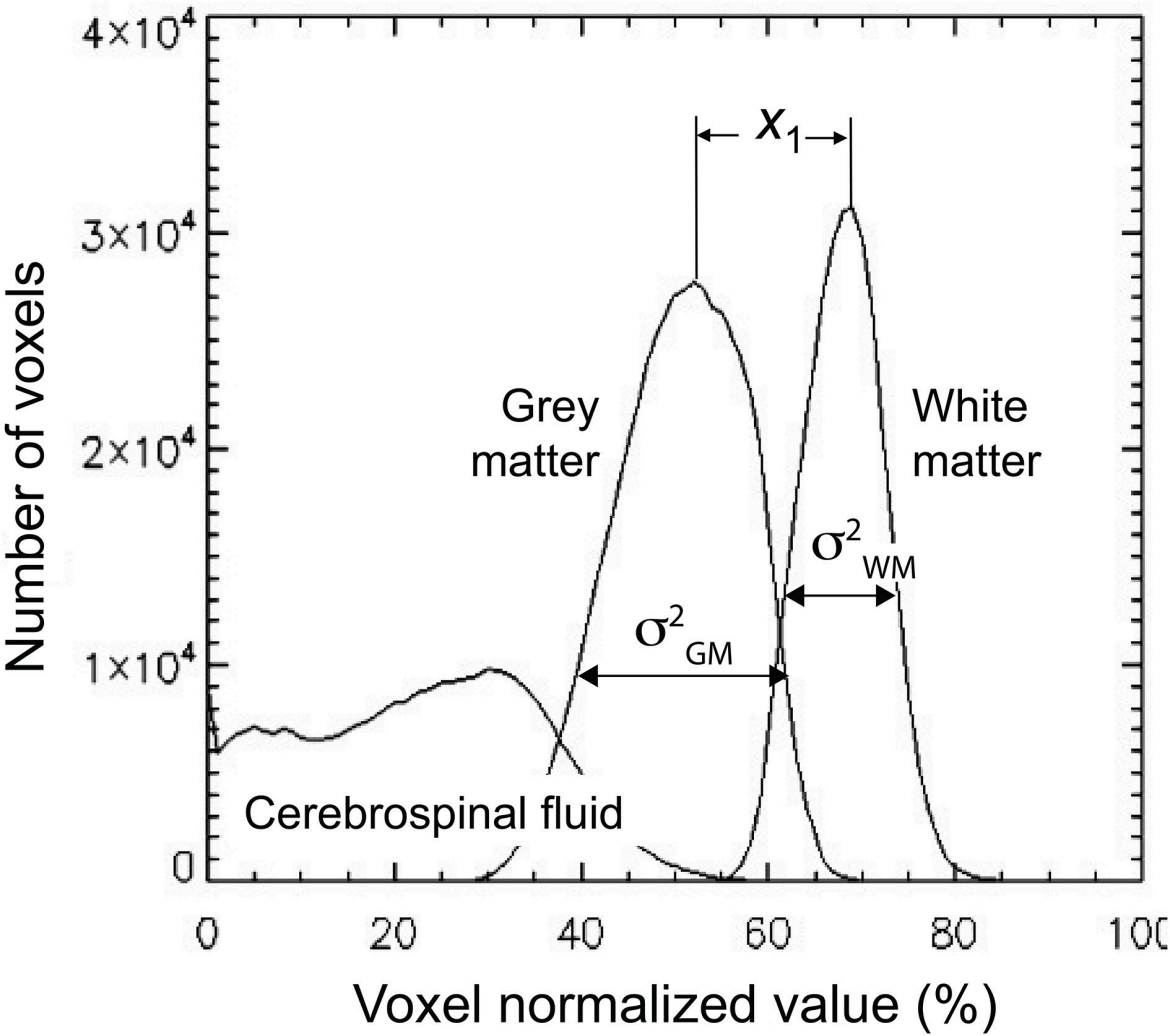
**The gw_t_score feature (VF3) is computed from the distribution of the gray and white matter class map histogram**. The difference in means of the two distributions is estimated to be *x*_1_ and the variance for each distribution is estimated as $\sigma_{GM}^{2}$ and $\sigma_{WM}^{2}$. These estimates are used to compute the gw_t_score given in Equation (4).

The third volumetric feature is a single statistic, denoted as *gw*_*t*_*score*, that quantified the contrast between gray and white matter. It is defined as follows. First a binary class map, i.e., a mask, was created for both white matter and gray matter using a threshold of 0.5. These masks were then multiplied voxelwise by the 3D-MRIs **I**_*w,i*_, to generate class map intensity maps **I**_*c,i*_. A histogram **VF1**_*c*_ was computed for each class in the same way as in Equation (1) by substituting **I**_*c,i*_ for **I**_*w,i*_. The profiles for the histogram are illustrated for a representative 3D-MRI in Figure 4. The mean ${\hat{\mu }}_{c}$, and variance ${\hat{\sigma }}_{c}^{2}$ of the two classes were estimated using the following formulas:

(3) $$\begin{array}{l} {\hat{\mu }}_{c}=\frac{1}{100}\sum_{I_{n}}VF1_{c}(I_{n})\\ {\hat{\sigma }}_{c}^{2}=\frac{1}{100}\sum_{I_{n}}[VF1_{c}(I_{n})-{\hat{\mu }}_{c}{]}^{2} \end{array}$$

where c = {white matter, gray matter}, and the sum was computed over the 100 stepping intensity values *I*_*n*_, defined for Equation (1). The difference between the means for each class was computed as $x_{1}={\hat{\mu }}_{WM}-{\hat{\mu }}_{GM}$ and the statistic *gw*_*t*_*score*, was computed as:

(4) $$\begin{array}{r} VF3=gw\text{\_}t\text{\_}score=\frac{x_{1}}{\sqrt{{\hat{\sigma }}_{GM}^{2}+{\hat{\sigma }}_{WM}^{2}}} \end{array}$$

This value was estimated for all 3D-MRIs as *VF*3 and used as the third volumetric feature in SVM.

#### 2.3.2. Artifact-specific features (ASF)

Artifact-specific features were defined to target the three most prominent artifacts: eye movement, ringing, and aliasing. As shown in Table 1, of the 3D-MRI volumes containing at least one type of artifact, 94% have, at least one of the three artifacts targeted. This motivated the search for features that target each of these artifacts. The artifact-specific features developed in this project were later combined as inputs into the SVM.

**Table 1:** **Distribution for the artifacts explored are presented here**.

**Artifact class**	**Number of images**	**Percent (%)**
No artifacts	665	46
Eye movement or ringing or aliasing	746	51
Other artifacts	46	3
Total	1457	100

*A total of 51% of all structural magnetic resonance images (3D-MRIs) contain at least one of the three artifacts explored. Nearly 94% of the 792 3D-MRIs containing at least one type of artifact are comprised of eye movement, ringing, or aliasing artifact*.

A *background-mask* located entirely outside the head was created for each individual subject to isolate and help quantify artifacts related to eye movement and ringing. The *background-mask* was created by combining the individual subject VBM-generated *background maps*, in the following way. First, **p**_*b,i*_, the individual subject VBM-generated *background maps*, were spatially normalized using *H*_*i*_:

(5) $$\begin{array}{r} p_{b,i\prime }\left(x\prime ,y\prime ,z\prime \right)=H_{i}p_{b,i}\left(x,y,z\right) \end{array}$$

Then, the individual normalized *background maps* **p**_b, i′_ were averaged to compute the *background ROI* at (*x*′, *y*′, *z*′):

(6) $$\begin{array}{r} p_{b,ROI}\left(x\prime ,y\prime ,z\prime \right)=\frac{1}{N}\overset{N}{\underset{i\prime =1}{\sum }}p_{b,i\prime }\left(x\prime ,y\prime ,z\prime \right) \end{array}$$

where *N* = 1457 is the number of subjects. The *background ROI* **p**_*b,ROI*_, was then transformed back to each native space using $H_{i}^{-1}$ to generate a *common background* **p**_*bc,i*_ for each individual subject *i* as:

(7) $$\begin{array}{r} p_{bc,i}\left(x,y,z\right)=H_{i}^{-1}p_{b,ROI}\left(x\prime ,y\prime ,z\prime \right) \end{array}$$

Finally, **p**_*bc,i*_ was thresholded at 0.5, to generate a mask named *background-mask* **I**_*b,i*_ for each individual subject *i*. The *background-mask* **I**_*b,i*_ was used for each subject to generate an *eye-mask* and a *ring-mask* to define features ASF1 and ASF2, respectively.

##### 2.3.2.1. ASF1—eye movement

**Figure 5:**
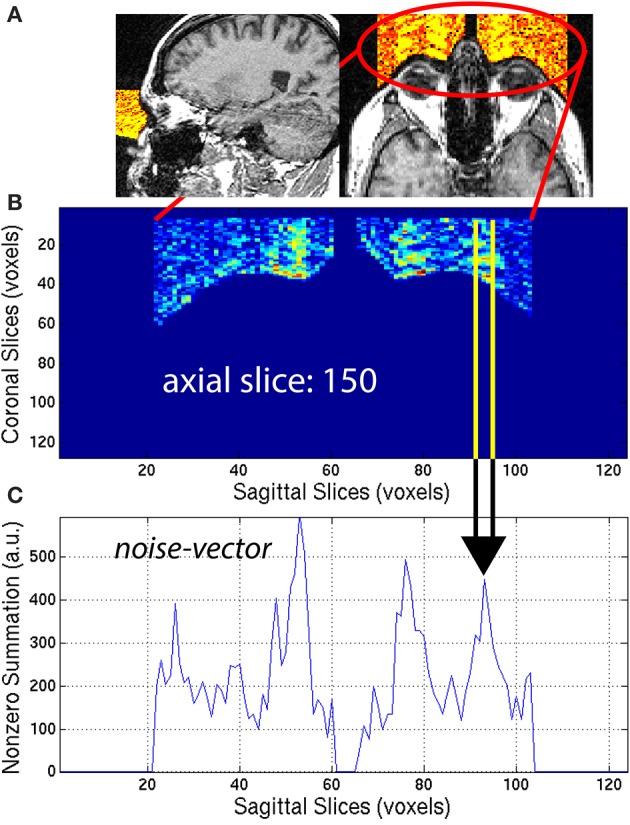
**(A)** An *eye-mask* is illustrated for a representative subject with noticeable eye movement artifact. **(B)** Each axial slice of the *eye-mask* was collapsed into a **(C)** *noise-vector*. The *noise-vector* is equal to the median, non-zero voxel of each column of voxels of the *eye-mask*, as in Equation (8). The feature ASF1 was computed as the max of the sum of the *noise-vector* as in Equation (9). This procedure is illustrated for a representative axial slice, *z* = 150.

Eye-movement generated artifacts consist of excess noise both inside the brain, and in front of the eyes. To quantify the amount of noise generated by eye movement, a region located directly in front of the eyes was masked. To that end, the location and dimensions of the eye sockets in normalized space (*x*′, *y*′, *z*′) were first manually estimated, then the location and dimensions were warped to native space (*x, y, z*), using the inverse transformation matrix $H_{i}^{-1}$. An *eye-mask* **I**_*eye,i*_ was then generated by taking the intersection of the *background-mask* **I**_*b,i*_ with the projection in the y-direction of the eye sockets. This automated procedure efficiently extracted the region in front of both eyes, for all subjects. The *eye-mask* is illustrated in yellow and orange colors to highlight the signal present in the *eye-mask* for a representative 3D-MRI in Figure 5A.

The *eye-mask* was used to compute ASF1, in the following way. First, for each axial slice *z* = *z*_0_, of the *eye-mask* **I**_*eye,i*_, the median of non-zero voxels was computed along the *y*-axis (coronal direction) to create a *noise-vector* **I**_*nv,i*_ along the *x*-axis (sagittal direction):

(8) $$I_{nv,i}(x,z_{0})=\underset{y}{\text{med}}\left[I_{eye,i}(x,y,z_{0})>0\right]$$

This procedure generated a *noise-vector* **I**_*nv,i*_ for each axial slice, *z*_0_. The median provides a stable measure of the noise and is not sensitive to spurious detection when compared to the maximum or arithmetic mean. This mathematical computation is illustrated in Figure 5 for one representative axial slice *z*_0_ = 150, of one representative subject, with visible noise related to eye movement. The *noise-vector* **I**_*nv,i*_ was summed along the *x*-axis, to subsequently define the feature ASF1 as the maximum over the axial slices *z*_0_:

(9) $$\begin{array}{r} \text{ASF1}=\underset{z_{0}}{\max}\underset{x}{\sum }I_{nv,i}\left(x,z_{0}\right) \end{array}$$

This feature was calculated for each subject *i* and used as ASF1 in the SVM classifier.

##### 2.3.2.2. ASF2—ringing

**Figure 6:**
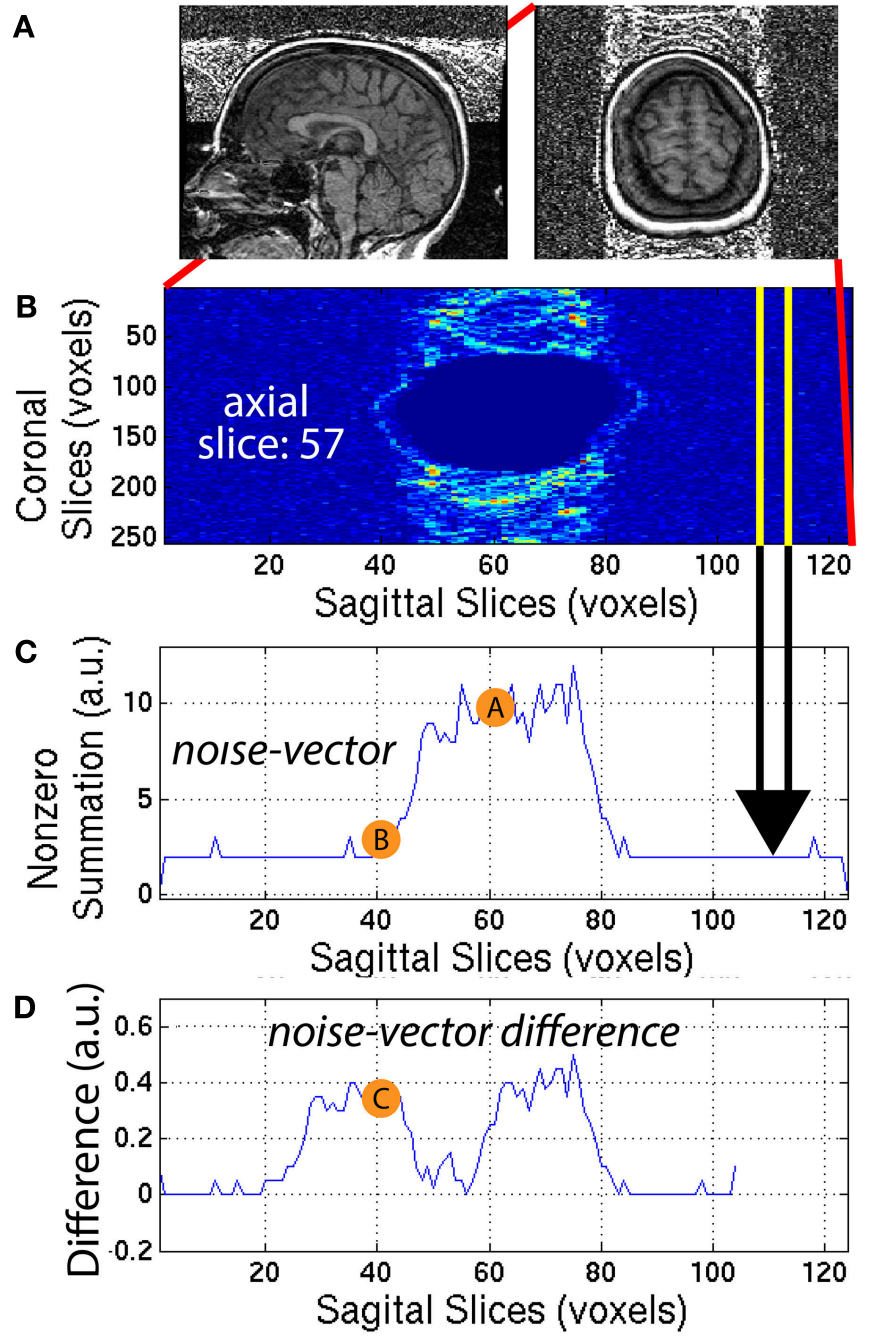
**(A)** A representative subject with noticeable ringing artifact is illustrated. **(B)** Each axial slice of the *ring-mask* was collapsed into a **(C)** *noise-vector*. The *noise-vector* is equal to the median non-zero voxel for each column of voxels of the *ring-mask*, as in Equation (8). **(D)** The *noise-vector difference* is equal the absolute value of the difference between shifted versions of the *noise-vector*, where $C=\frac{\left|A-B\right|}{20}$, as in Equation (10). The feature ASF2 was computed as the max of the sum of the *noise-vector difference* as in Equation (11). This procedure is illustrated for a representative axial slice, *z* = 57.

Ringing artifacts contain noise around the top of the brain and outside the head as well. To quantify the ringing artifact, the *background-mask* **I**_*b,i*_ was used to create a *ring-mask* **I**_*ring,i*_ centered on the top of the brain and entirely outside the head. A *ring-mask* **I**_*ring,i*_, generated by this automated procedure, is illustrated in Figure 6A for a representative 3D-MRI with noticeable ringing artifact. The *ring-mask* **I**_*ring,i*_, was bounded at the bottom to avoid interference from eye movement artifacts and at the top to avoid aliasing along the axial direction.

The *ring-mask* **I**_*ring,i*_ was used to compute ASF2, as follows. For each axial slice *z* = *z*_1_, of the *ring-mask* **I**_*ring,i*_, a *noise-vector* **I**_*nv,i*_, was computed as in Equation (8). Then, a *noise-vector difference* **I**_*nvd,i*_, was computed as the absolute difference between shifted versions of the *noise-vector* **I**_*nv,i*_:

(10) $$\begin{array}{r} I_{nvd,i}\left(x,z_{1}\right)=\frac{\left|I_{nv,i}\left(x+20,z_{1}\right)-I_{nv,i}\left(x,z_{1}\right)\right|}{20} \end{array}$$

where *x* = [1, 104] is truncated by the window size = 20 voxels. A window size of 20 voxels provided the necessary distance along the *x*-axis, to define a feature that robustly quantified the noise, when compared to the background signal. This mathematical computation is illustrated in Figure 6 for one representative axial slice *z*_1_ = 57, of one representative subject. The *noise-vector difference* **I**_*nvd,i*_ was summed along the *x*-axis, to subsequently define the feature ASF2 as the maximum over all axial slices *z*_1_:

(11) $$\begin{array}{r} \text{ASF2}=\underset{z_{1}}{\max}\underset{x}{\sum }I_{nvd,i}\left(x,z_{1}\right) \end{array}$$

This feature was calculated for each subject *i* and used as ASF2 in the SVM classifier.

##### 2.3.2.3. ASF3—aliasing

**Figure 7:**
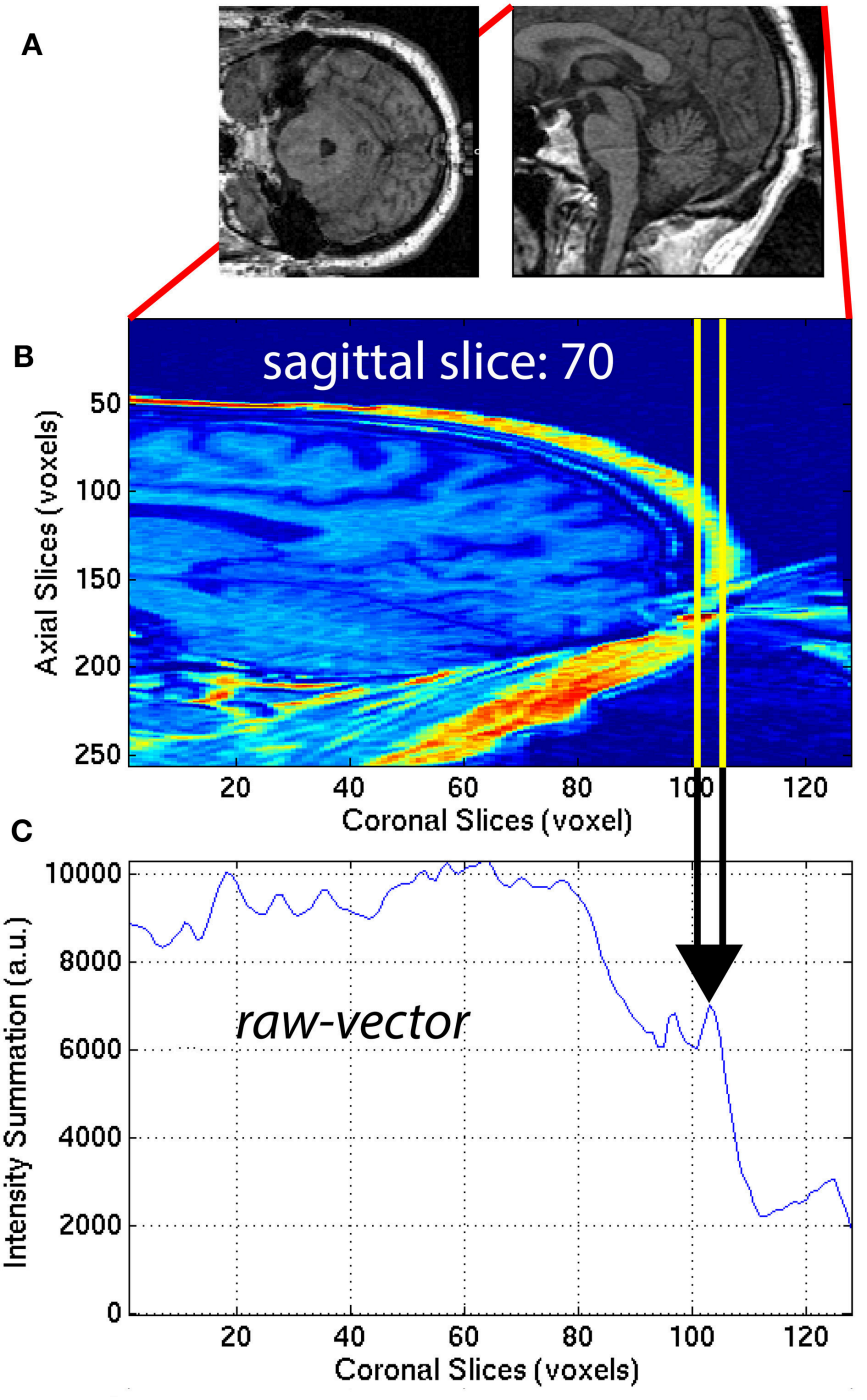
**(A)** Axial and sagittal slices illustrate how the nose of the subject can wrap around the image and cause an artifact on the back of the brain. The 30 sagittal slices, *x*_1_ = [48, 77] centered on the midline (*x*_*m*_ = 62) were selected to quantify the aliasing artifact. For each **(B)** sagittal slice, a **(C)** *raw-vector* was computed by summing along the *z*-axis (axial direction), as in Equation (12). The minimum along the *y*-axis (coronal direction) was computed to define ASF3 as the maximum over the 30 sagittal slices *x*_1_, as in Equation (13).

The aliasing artifact causes the nose and other facial features to contaminate the posterior part of the brain, as illustrated in Figure 7A. To quantify the amount of aliasing, the region located posterior to the head was used. For each of the 30 sagittal slices *x* = *x*_1_ = [48, 77] centered on the midline *x*_*m*_ = 62 of the 3D-MRI **I**_*w,i*_, the summation along the *z*-axis (axial direction) was computed to create a *raw-vector* **I**_*rv,i*_:

(12) $$\begin{array}{r} I_{rv,i}\left(x_{1},y\right)=\underset{z}{\sum }I_{w,i}\left(x_{1},y,z\right) \end{array}$$

This procedure generated a *raw-vector* **I**_*rv,i*_ for each sagittal slice, *x*_1_. This mathematical computation is illustrated in Figure 7 for one representative sagittal slice *x*_1_ = 70, of one representative subject, with noticeable nose aliasing. The minimum value of the *raw-vector* **I**_*rv,i*_ along the y-axis (coronal direction) was computed, and defined ASF3 as the maximum over the 30 sagittal slices, *x*_1_:

(13) $$\begin{array}{r} \text{ASF3}=\underset{x_{1}}{\max}\underset{y}{\min}I_{rv,i}\left(x_{1},y\right) \end{array}$$

The minimum value along the y-axis was computed, instead of the median. The motivation for this distinction can be understood by computing ASF3 for 3D-MRI volumes representing three different scenarios: (1) no aliasing, (2) aliasing but nose does not touch back of head, and (3) aliasing and nose gets overlaid into the head. In both cases (1) and (2), if the nose is clearly separated from the back of the head, ASF3 will be close to the background value. Otherwise in case (3), ASF3 will be closer to the intensity value of the nose. The lower the value for ASF3 indicates the nose is more separated from the back of the head, resulting in a metric that quantifies separation. This feature was calculated for each subject *i* and used as ASF3 in the SVM classifier.

### 2.4. SVM classification

**Figure 8:**
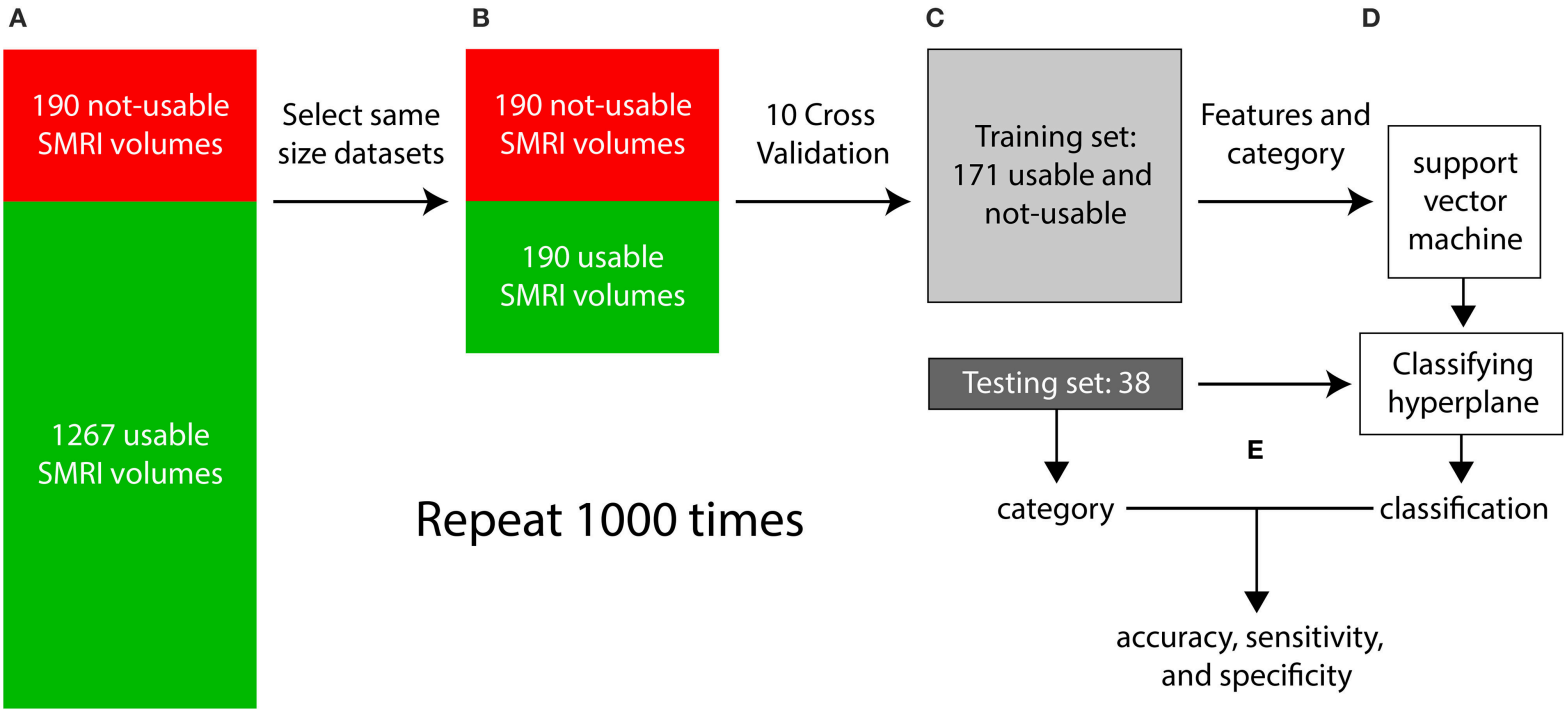
**The flow of the classification method is illustrated above**. **(A)** First the 3D-MRI volumes are categorized as usable and unusable as explained in Figure 2. **(B)** The same number of usable 3D-MRIs were chosen randomly to create two groups of the same length. **(C)** The two groups were subdivided using 10-fold cross validation, with 90% of the datasets in the training and 10% in the testing. **(D)** The features from the training group along with their category, determined by visual inspection were used as input into the support vector machine (SVM) to generate a classifying hyperplane. The hyperplane was then used along with the features of the testing set to classify the testing 3D-MRIs as usable or not-usable. **(E)** This categorization is compared to the visually inspected category to compute an accuracy, specificity, and sensitivity. The entire procedure was repeated 1000 times to account for the variability in the usable datasets.

Each iteration of the supervised classification procedure consisted of a training stage and a testing stage and is illustrated in Figure 8. In step (a), visual inspection categorized the dataset into 1267 “usable” 3D-MRIs and 190 “not-usable” 3D-MRIs. In (b), 190 of the 1267 “usable” 3D-MRI volumes were selected randomly to generate two equal size “usable” and “not-usable” datasets to reduce bias in the classification procedure. In step (c), a 10-fold cross validation procedure was used to generate a training set and a testing set composed of 90% and 10% of the data, respectively. In step (d), the features of the training set along with the visually inspected category were used as input into the SVM to generate a classifying hyperplane. In step (e) the hyperplane was then used to classify the features from the testing set as “usable” or “not-usable.” This classification was then compared to the visually inspected category in order to compute accuracy, sensitivity, and specificity of the classification procedure. This entire procedure was repeated 1000 times in order to select different subsets of the “usable” 3D-MRI volumes and account for variability in the dataset.

## 3. Results

First, the reliability of the artifact-specific features (ASFs) was investigated to assess how well these features captured the artifacts. Second, SVM performance was analyzed by computing the accuracy of the correctly predicted 3D-MRI when compared to the visually inspected category.

### 3.1. The performance of the artifact-specific features

ASFs were developed using 3D-MRI volumes that were tagged yellow, after the stage I of the visual inspection procedure (Figure 2). We hypothesized that if the ASFs have the sensitivity to quantify the artifacts from these yellow-tagged 3D-MRI volumes, then the features should indicate that green-tagged 3D-MRI volumes are free from artifacts, and red-tagged 3D-MRI volumes are comprised of one or more heavy artifacts. The sensitivity of each ASF was assessed within the yellow-tagged 3D-MRI by comparing with the visual-inspection scale of heavy, moderate, slight, or none.

#### 3.1.1. ASF1—eye movement

**Figure 9:**
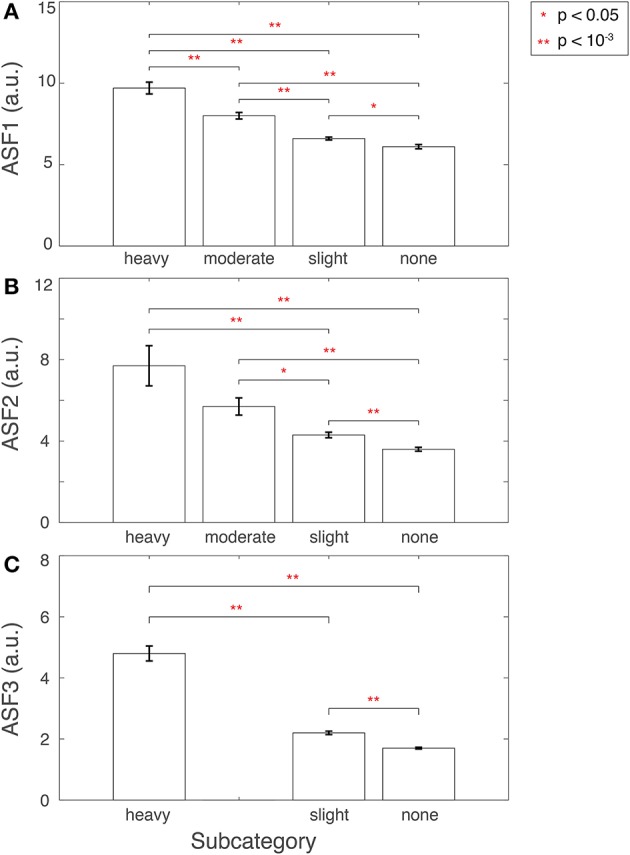
**Features (A)** ASF1, **(B)** ASF2, and **(C)** ASF3 were computed for 3D-MRI volumes tagged yellow and divided into corresponding subcategories: heavy, moderate, slight, and none. The distributions for each ASF are summarized above for each subcategory with an arithmetic mean, and the standard error of the mean. Corresponding *p*-values were computed based on Student's two-tailed *t*-test between each pair of subcategories. A *p* < 0.05 was used to determine if the distributions were statistically different between subcategories and are denoted with a red ^*^, while *p* < 10^−3^ are denoted with a red ^**^.

ASF1 was compared to the 4° rating scale by summarizing the distributions as a bar plot in Figure 9A. The subcategory from the visual inspection procedure gets worse in the 3D-MRIs, i.e., from none to heavy, as the value for ASF1 correspondingly increases. A *p*-value, based on Student's two tailed *t*-test, was computed to determine if the subcategories were statistically different from each other. All pairs of subcategories resulted in *p* < 0.05, denoted by a ^*^, while *p* < 10^−3^ were denoted with ^**^.

Furthermore, another look at the 3D-MRI volumes computed to have high ASF1 but originally labeled by the human experts to have slight or no eye movement related artifacts, in fact, revealed to have heavy eye movement artifacts.

#### 3.1.2. ASF2—ringing

ASF2 was compared to the 4° rating scale by summarizing the distributions as box plot in Figure 9B. The subcategory from the visual inspection procedure gets worse in the 3D-MRIs, i.e., from none to heavy, as the value for the ASF2 correspondingly increases. A *p*-value, based on Student's two tailed *t*-test, was computed to determine if the subcategories were statistically different from each other. All pairs of subcategories resulted in *p* < 0.05, denoted by a ^*^, while *p* < 10^−3^ were denoted with ^**^. The heavy-moderate combination was determined to be statistically non-significant.

In addition, a second visual inspection of the 3D-MRI with a high value of ASF2 and labeled by human experts to have slight or no ringing, disclosed that these 3D-MRI volumes in reality had heavy ringing artifact.

#### 3.1.3. ASF3—aliasing

Finally, ASF3 was compared to the 4° rating scale by summarizing the distributions as a bar plot in Figure 9C. There were no yellow-tagged images labeled to have moderate aliasing. The subcategory from the visual inspection procedure gets worse in the 3D-MRIs, i.e., from none to heavy, as the value for the ASF3 mean correspondingly increases. A *p*-value, based on Student's two-tailed *t*-test, was computed to determine if the subcategories were statistically different from each other. All pairs of subcategories resulted in a *p* < 10^−3^ denoted by ^**^.

### 3.2. SVM classification performance

**Figure 10:**
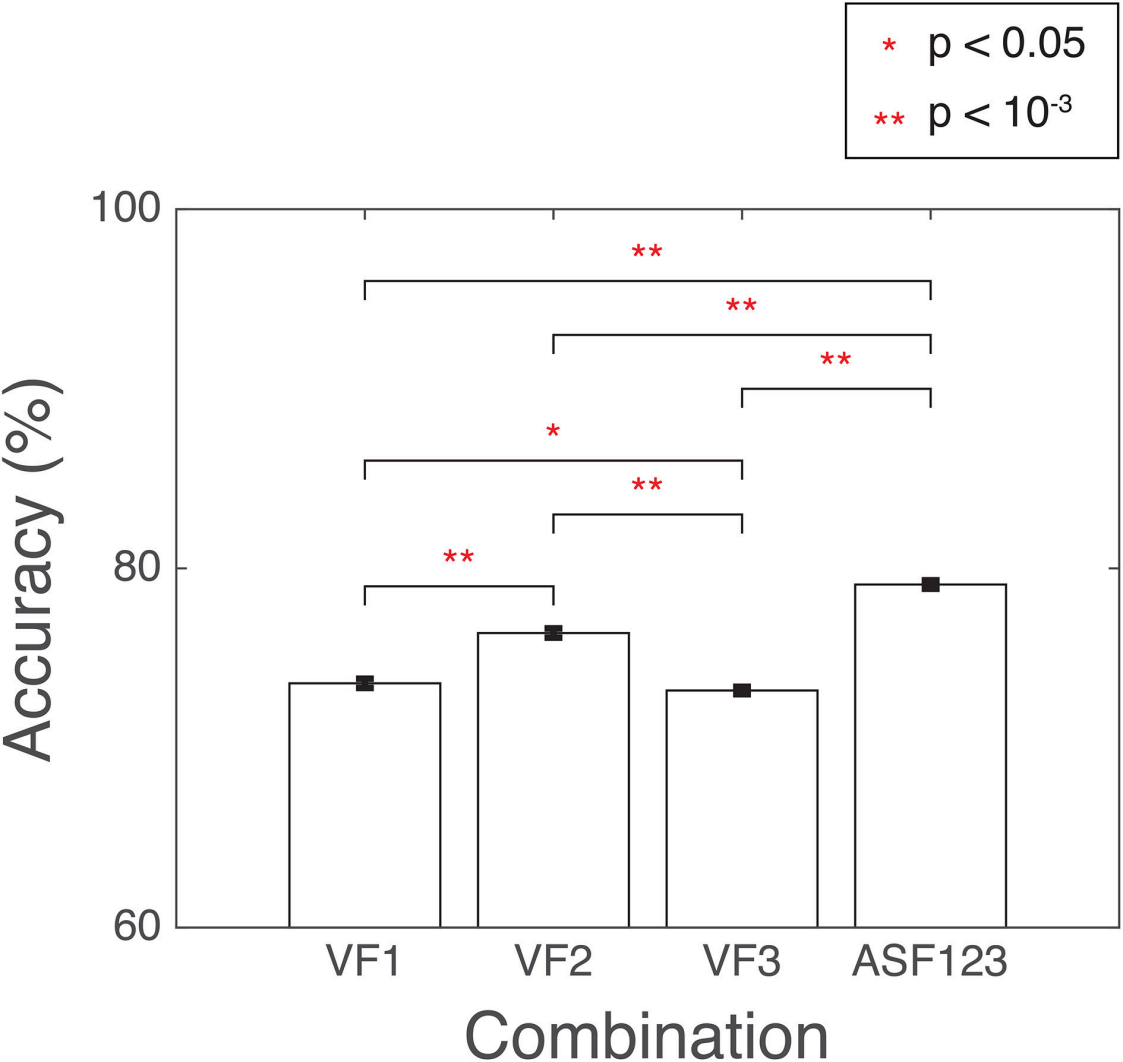
**SVM performance is reported here for the combination of features with the highest accuracy, summarized with a mean and the standard error of the mean, reported as a %**. Accuracy was computed as the number of SVM correctly classified when compared to the visual-inspection category. Corresponding *p*-values were computed based on Student's two-tailed *t*-test between each pair of combinations. A *p* < 0.05 was used as threshold to determine if the performance was statistically different between combinations and are denoted with a red ^*^, while *p* < 10^−3^ are denoted with a red ^**^.

Several combinations of the developed features were used as input into SVM. The combination with the highest accuracy was chosen to be the winning set. The accuracy was computed as the percentage of correctly classified 3D-MRI volumes as compared the visual-inspection category. The combinations yielding the highest accuracies are displayed in Figure 10. Other combinations that resulted in lower accuracies indicated that the SVM was being incorrectly trained and that a less discriminate hyperplane was being generated. More information was not necessarily helpful and resulted in more misclassified 3D-MRIs. A statistical comparison across the different combinations, based on Student's two-tailed *t*-test, was conducted. Corresponding *p* < 0.05 are denoted by a ^*^, while *p* < 10^−3^ are denoted with ^**^. This indicates that the artifact-specific features (ASF) outperformed any other combination of volumetric features (VF). The sensitivity and specificity of the SVM were computed to further explore the performance of the classifier. Sensitivity was defined to be the number of 3D-MRI volumes correctly identified to be “not-usable” and specificity was defined to be the number of 3D-MRI volumes correctly identified to be “usable.” For the ASF combination, the sensitivity and specificity were equal to 70.1 and 88.2%, respectively.

**Figure 11:**
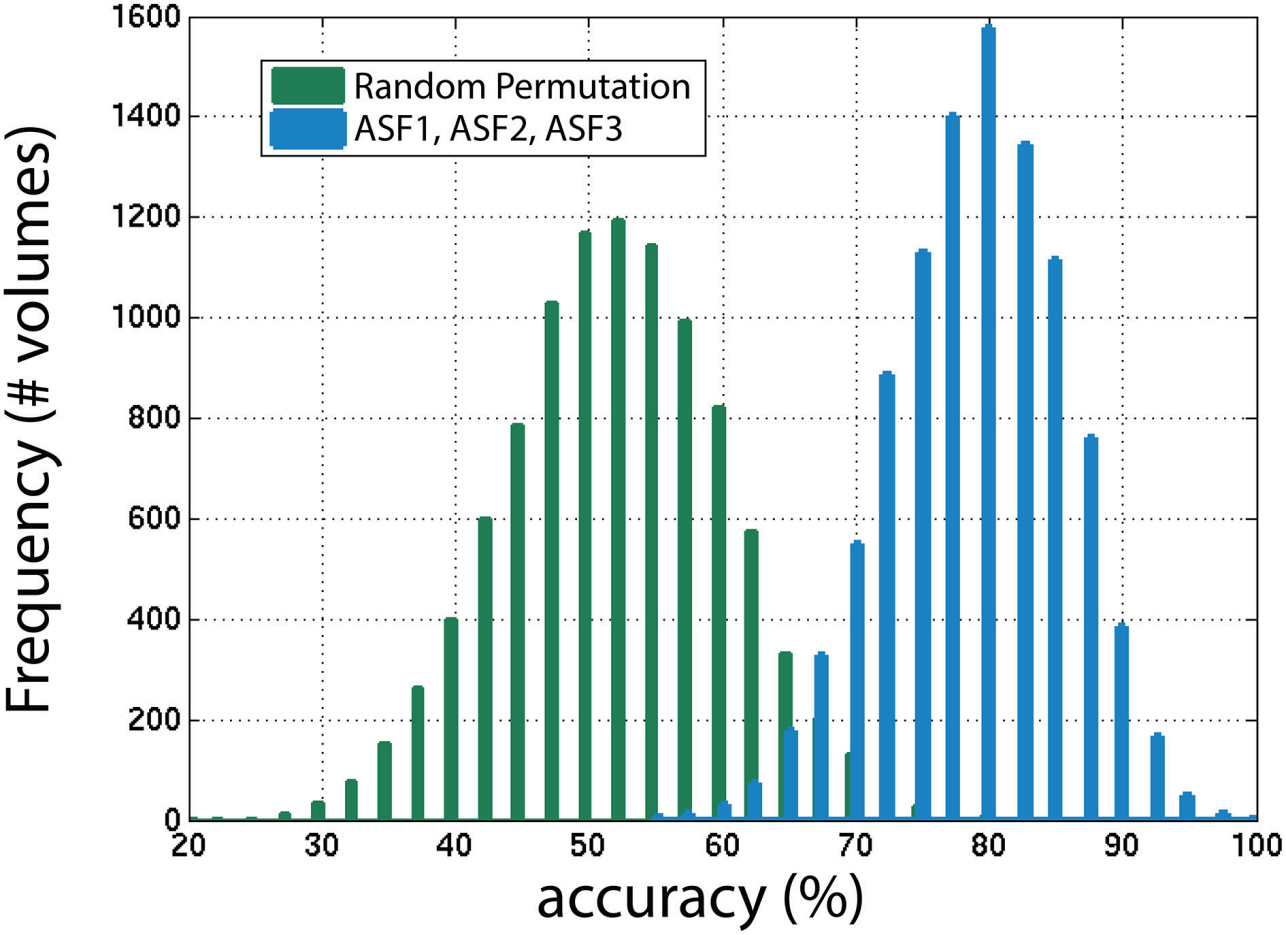
**The distribution of accuracies generated from 1000 iterations with SVM for the winning combination of features (ASF1, ASF2, and ASF3) vs. random permutation of the categories in the training portion**. In the random permutation, the corresponding category labels were flipped randomly to incorrectly train SVM with the wrong information. This procedure is similar to “set-level interference” used in functional brain imaging (Friston et al., 1996), as described in the text.

Next, the distribution of the ASF results were compared to a random permutation, shown in Figure 11, to check if the ASF results are significantly higher than chance. In the random permutation iterations, everything was kept identical to the SVM classification except that, in the training portion, the “usable” and “not-usable” labels were randomly rearranged to each 3D-MRI. This ensured that the classifier was not getting properly trained and the hyperplane generated was not based on relevant data. This comparison is similar to “set-level interference,” an approach used in functional brain imaging (Friston et al., 1996). From the 10,000 permutations, only six were above the average obtained by the classifier when using the three artifact dependent features. This analysis ensured that the results obtained were statistically significant (*p* < 0.001). This is in agreement to the Student's two sample, two-tailed *t*-test between the distribution of the SVM classification using the ASF results and the random permutation. A *t*-value of 80.1 was computed, corresponding to a *p* < 10^−4^, making the ASF results statistically higher than chance.

## 4. Discussion

Compared to a univariate quality assessment approach that generates a single number, the multivariate approach (together with the global and ROI automated image quality features) presented in this paper is more informative as it provides details that categorize, localize, and quantify the extent of noise in the data. These parameters are key tools for assessing the quality of 3D-MRI in a neuroimaging database, where the brain 3D-MRI volumes are indexed and can be queried according to the artifact type. Moreover, since the features used in the classifier are regional, the affected regions can be highlighted in an automated plugin added to a 3D imaging viewer. This plugin can then be used to improve the efficiency of the quality assessment procedure of 3D-MRIs through visual inspection by helping investigators to focus in on the problematic regions. Such a task is not possible with a global feature that is estimated from the entire volume.

The categories generated by visual-inspection were used as the gold standard when developing the automated classification procedure. In visual-inspection, an investigator first looks for regions that may contain artifacts, visually rates the level of artifact as heavy, moderate, slight or none, and then makes an overall conclusion with an appropriate label (red, yellow, green). The features that produced the highest accuracy were based on first quantifying the artifacts, then using the machine-learning algorithm to classify the 3D-MRIs as usable or non-usable. The multivariate classifier estimates a discriminate hyperplane, by incorporating all features rather than attempting to classify by each feature individually. Individual classification would lead to incongruent information resulting in lower performance accuracy. The winning automated-procedure emulates most closely the visual-inspection procedure, by first objectively quantifying each artifact, then estimating the most discriminative hyperplane to classify each 3D-MRI as usable or not-usable. This could potentially explain why the volumetric features such as the histogram based features or the gw-t-score did not perform as well as the artifact-specific features.

The SVM classifying approach was able to correctly classify 70.1% of the 3D-MRI that were assigned “not-usable” based on investigator-based visual inspection. In addition, the SVM approach was able to classify 88.2% of the 3D-MRI that were assigned “usable” based on investigator-based visual inspection. Based on these sensitivity and specificity estimates, the accuracy of our SVM approach and automated image quality features was computed to be around 80%. The remainder of this section is devoted to identification and discussion of the strengths and weaknesses in the current approach and possible reasons for the discrepancy between sensitivity and specificity measures, with the goal of providing areas to further improve the current methodology.

### 4.1. Resolve the misclassified datasets

The reasons for misclassification can either be related to human error, SVM classification error, or a combination of the two. Human error misclassification is an important motivation for this study and can occur for a number of reasons. Visual inspection of structural 3D-MRI volumes in large databases may require that this process be shared by a number of experts rather than an individual investigator, which can introduce inter-rater variability in how the 3D-MRI volumes are labeled. Even if the task were assigned to a single investigator, intra-individual variability though probably less than inter-individual variability is still an issue that needs to be addressed.

Misclassification of a 3D-MRI due to machine error suggests the developed methods can be further refined, to improve performance. One problem, that came to light through this work is when an artifact is not present in the air background and exists outside the targeted region. The ringing artifact problem, for example, can be present completely inside the brain and not cause any changes in the background part of the image. The value for ASF2, in this case, would be small making the SVM classifier assign minimal or no ringing artifacts, when in reality there is ringing artifact corrupting the 3D-MRI. In order to address this issue, additional image processing tools would need to be developed to distinguish this artifact from the brain.

The power of the artifact-specific features is the automated and flexible nature of these metrics. ASF3 was developed and automated to target aliasing in the coronal direction. However, aliasing can also be present in the sagittal and axial directions. In some of the 3D-MRIs of the dataset analyzed, there was aliasing in the axial direction, with 2–3 axial neck slices wrapping around to the top. The *ring-mask* used to define Equation (11) was bounded at the top to avoid aliasing in the axial direction. While ASF3 was developed to capture aliasing in the coronal direction, this feature can easily be implemented to measure the extent of aliasing in all directions.

While the artifact-specific features were developed to target artifacts found in 3D-MRI volumes acquired using a spoiled gradient recalled (SPGR) sequence, they can be used for assessing 3D-MRIs acquired using other pulse sequence. To extract meaningful features, investigators can follow a two-step approach: (1) identify recurring artifacts specific to the acquisition pulse sequence and (2) adapt the automated artifact-specific features to target the most prominent artifacts.

### 4.2. Overcome limits of the current approach

#### 4.2.1. Develop additional features: ASF4, ASF5, and ASF6

As illustrated in Table 1, 51% of 3D-MRI volumes contain one of the three most prominent artifacts: eye movement, ringing, or aliasing. A subset of these also contains additional artifacts, namely grainy images, and artifacts from head movement and teeth fillings. A small number of misclassified 3D-MRI volumes of around 5% can be attributed to the lack of features describing these artifacts. Future work in this project would attempt to describe these less prominent artifacts by developing additional artifact-specific features: ASF4, ASF5, and ASF6.

#### 4.2.2. Develop dedicated features to capture contrast and bias related issues

In the current study, the proposed objective metrics were developed to emulate the visual inspection process. It should therefore be noted that even if our proposed method worked with 100% accuracy, it still could translate into a limitation of this study. This is because visual inspection, besides being a subjective and inconsistent process, is also limited by the inability of humans to pick up subtle artifacts. For instance, the visual inspection process may not identify 3D-MRIs that cause automatic segmentation algorithms to fail due to contrast or bias artifact issues in the MR images. This is important in algorithms that have multiple steps and extract fine detailed measurements, like Freesurfer (Fischl, 2012). This limitation can be potentially addressed in the future by: (1) using an improved gold standard that is based not only on human visual inspection, but also on feedback from automated algorithms such as Freesurfer, and (2) developing dedicated features that can capture the artifacts causing automated algorithms to fail and are more sensitive than human-raters (Gardner et al., 1995), and are able to detect contrast or bias related issues.

### 4.3. Calculate the confidence interval for classification

The automated package presented in this paper can potentially be used for pre-screening purposes in order to cut down the amount of work and time spent rating the 3D-MRI visually by investigators. Toward this end, a confidence level for each classification result can be computed so that if the classification is below a certain confidence level, an investigator can further visually inspect the dataset for further clarification.

## 5. Conclusion

A novel method to automatically assess the quality of images using a multivariate classifier has been proposed. This is the first study that uses a multidimensional machine-learning algorithm based on automated regional extracted features to automate quality assessment of structural magnetic resonance images (MRI). Compared to a univariate quality assessment approach that generates a single number, the approach presented in this paper is more informative as it provides details that categorize, localize and quantify the extent of noise in the data. By breaking the problem down into smaller problems, features have been developed that individually quantify each of the artifacts that can be used as inputs into the SVM classifier. These parameters are key tools for assessing the quality of 3D-MRI in a neuroimaging database where the brain images are indexed and can be queried according to the different types of artifacts. Moreover, since the features used in the classifier are regional and localized, affected regions can be automatically marked in an imaging viewer, improving the efficiency for the human visual inspection procedure, a task not possible with a feature that is estimated from the entire 3D-MRI. The accuracy is close to 80%, and can increase with additional work to include more features to account for the other artifacts not yet explored.

## Author contributions

RP, XC, VM, and QL made substantial contribution to the concept and design of the work. KB, JC, DW, VM, BV, AG, and EX made substantial contribution to the acquisition of data for the work. RP, XC, AB, HL, BV, AG, and VM made substantial contribution to the analysis and interpretation of data for the work. RP, XC, and VM drafted the manuscript critically. All co-authors revised the manuscript critically for important intellectual content. All co-authors approved the final version to be published. All co-authors agreed to be accountable for all aspects of the work ensuring the accuracy and integrity of the work have been appropriately investigated and resolved.

## Funding

This research was supported by (1) the Intramural Research Program of the National Institute of Mental Health, NIH, Bethesda, MD 20892, USA, (2) the Office of Science Management and Operations (OSMO) of the NIAID, Bethesda, MD 20892, USA, and (3) the Lieber Institute for Brain Development, Johns Hopkins Medical Campus, Baltimore, MD 21205, USA. RP, Ph.D. was supported (in part) by Award Number R25GM083252 from the National Institute of General Medical Sciences.

### Conflict of interest statement

The authors declare that the research was conducted in the absence of any commercial or financial relationships that could be construed as a potential conflict of interest. Material has been reviewed by the Walter Reed Army Institute of Research. There is no objection to its presentation and/or publication. The opinions or assertions contained herein are the private views of the author, and are not to be construed as official, or as reflecting true views of the Department of the Army or the Department of Defense.

## Footnotes

^1^3D-MRI and 3D-MRI volume are used interchangeably throughout the manuscript.

## References

[B1] Ashburner J. Friston K. J. (2000). Voxel-based morphometry, the methods. Neuroimage 11, 805–821. 10.1006/nimg.2000.0582 10860804

[B2] Ashburner J. Friston K. J. (2005). Unified segmentation. Neuroimage 26, 839–851. 10.1016/j.neuroimage.2005.02.018 15955494

[B3] Burges C. J. (1998). A tutorial on support vector machines for pattern recognition. Data Mining Know. Discov. 2, 121–167.

[B4] Cheng X. Pizarro R. Tong Y. Zoltick B. Luo Q. Weinberger D. R.. (2009). Bio-swarm-pipeline: a light-weight, extensible batch processing system for efficient biomedical data processing. Front. Neuroinform. 3:35. 10.3389/neuro.11.035.2009 19847314 PMC2763889

[B5] Fischl B. (2012). Freesurfer. NeuroImage 62, 774–781. 10.1016/j.neuroimage.2012.01.021 22248573 PMC3685476

[B6] Friston K. J. (2003). Statistical parametric mapping, in Neuroscience Databases, ed Kötter R.(New York, NY: Springer), 237–250.

[B7] Friston K. J. Holmes A. Poline J.-B. Price C. J. Frith C. D. (1996). Detecting activations in pet and fmri: levels of inference and power. Neuroimage 4, 223–235. 10.1006/nimg.1996.0074 9345513

[B8] Gardner E. A. Ellis J. H. Hyde R. J. Aisen A. M. Quint D. J. Carson P. L. (1995). Detection of degradation of magnetic resonance (MR) images: comparison of an automated MR image-quality analysis system with trained human observers. Acad. Radiol. 2, 277–281. 10.1016/S1076-6332(05)80184-9 9419562

[B9] Goldman A. L. Pezawas L. Mattay V. S. Fischl B. Verchinski B. A. Chen Q.. (2009). Widespread reductions of cortical thickness in schizophrenia and spectrum disorders and evidence of heritability. Arch. Gen. Psychiatry 66, 467–477. 10.1001/archgenpsychiatry.2009.24 19414706 PMC2719488

[B10] Jubault T. Gagnon J.-F. Karama S. Ptito A. Lafontaine A.-L. Evans A. C.. (2011). Patterns of cortical thickness and surface area in early parkinson's disease. Neuroimage 55, 462–467. 10.1016/j.neuroimage.2010.12.043 21184830

[B11] Magnotta V. A. Friedman L. Birn F. (2006). Measurement of signal-to-noise and contrast-to-noise in the fbirn multicenter imaging study. J. Digit. Imaging 19, 140–147. 10.1007/s10278-006-0264-x 16598643 PMC3045184

[B12] Mortamet B. Bernstein M. A. Jack C. R. Jr. Gunter J. L. Ward C. Britson P. J.. (2009). Automatic quality assessment in structural brain magnetic resonance imaging. Magn. Reson. Med. 62, 365–372. 10.1002/mrm.21992 19526493 PMC2780021

[B13] Sabuncu M. R. Desikan R. S. Sepulcre J. Yeo B. T. Liu H. Schmansky N. J.. (2011). The dynamics of cortical and hippocampal atrophy in Alzheimer disease. Arch. Neurol. 68, 1040–1048. 10.1001/archneurol.2011.167 21825241 PMC3248949

[B14] Vapnik V. (2013). The Nature of Statistical Learning Theory. New York, NY: Springer Science & Business Media.

[B15] Woodard J. P. Carley-Spencer M. P. (2006). No-reference image quality metrics for structural MRI. Neuroinformatics 4, 243–262. 10.1385/NI:4:3:243 16943630

